# Case Report: Behavioral Disorder Following Hemispherotomy: A Valproate Effect?

**DOI:** 10.3389/fneur.2021.764376

**Published:** 2021-11-30

**Authors:** Konstantin L. Makridis, Sebastian Triller, Deniz A. Atalay, Christine Prager, Christian E. Elger, Angela M. Kaindl

**Affiliations:** ^1^Charité – Universitätsmedizin Berlin, Department of Pediatric Neurology, Berlin, Germany; ^2^Charité – Universitätsmedizin Berlin, Center for Chronically Sick Children, Berlin, Germany; ^3^Charité – Universitätsmedizin Berlin, Institute of Cell- and Neurobiology, Berlin, Germany; ^4^Beta Neurology – Competence Center for Epilepsy, Bonn, Germany

**Keywords:** drug resistant epilepsy, pediatrics, behavioral problems, children, valproic acid, epilepsy surgery, hemispherotomy

## Abstract

**Background:** Hemispherotomy is an epilepsy surgery procedure applied to cure particularly pharmacorefractory lesional epilepsy due to unihemispheric pathologies. Such a disconnection of an entire hemisphere is followed by reorganizational processes.

**Methods:** We describe an acute aggravation of behavioral problems following a hemispherotomy in a patient treated with valproic acid, which subsided once valproate was discontinued.

**Results:** A 9-year-old boy with drug-resistant epilepsy caused by the residua of a perinatal stroke treated for several years with valproic acid and lamotrigine underwent hemispherotomy. Shortly after surgery, minimal preoperative behavioral problems intensified dramatically, and aggression occurred as a new symptom. Assuming a correlation between valproate treatment and the postoperative altered neuronal network, we tapered off valproate. The behavioral problems decreased in intensity with the reduction of valproate dose and disappeared after drug discontinuation.

**Conclusion:** We describe severe behavioral problems after hemispherotomy that subsided when valproate was tapered off. While we cannot rule out a spontaneous correction of a post-hemispherotomy network dysregulation, our report raises awareness to possible altered effects of the anticonvulsant valproic acid parallel to reorganizational processes after hemispherotomy.

## Introduction

Epilepsy is one of the most common neurologic diseases in childhood and adolescence (1). Antiseizure medications (ASMs) are the primary choice of treatment. However, in about one-third of affected individuals, they fail to achieve seizure freedom (2). In patients with drug-resistant structural epilepsy, the possibility of epilepsy surgery should be considered. Children with unilateral hemispheric lesions can profit from hemispherotomy. Several studies have illustrated functional reorganization after such an anatomic disconnection of a whole hemisphere. Here, we describe an aggravation of behavioral problems following hemispherotomy in a child treated with valproate (VPA). The behavioral problems subsided when VPA was discontinued. We suggest a possible altered anticonvulsant drug effect at a time when network changes likely occur.

## Case

**Figure 1:**
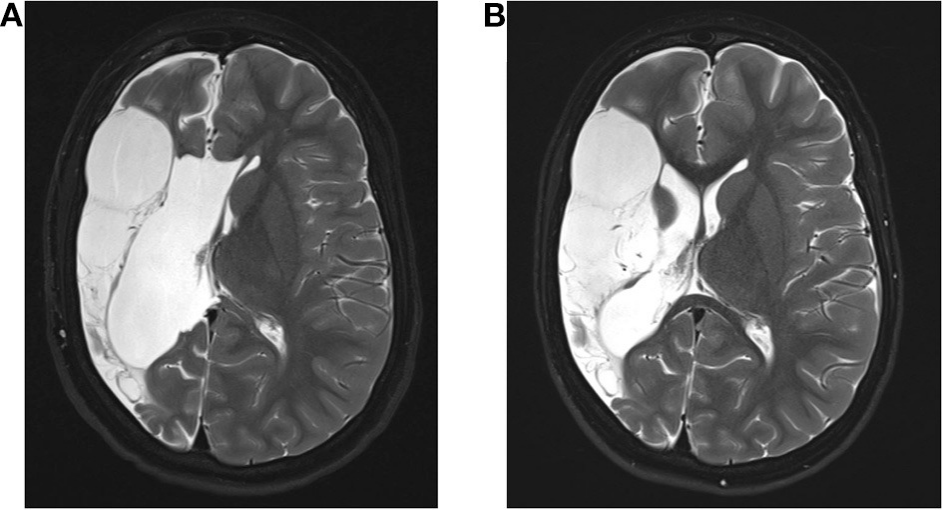
Magnetic resonance imaging (MRI) of the patient. **(A)** Shown is the preoperative MRI of the 2; 10 year old patient. **(B)** Normal MRI of the patient 6 months after surgery showing the malacic transformed medial infarct on the right side with no evidence of hemorrhage, infarction, or liquor circulatory disturbances.

A 9-year-old boy was outborn at term without complications as the first child of non-consanguineous and healthy parents after a complicated course of pregnancy (vaginal bleeding on gestational week 12, gestational diabetes). Postpartum weakness of the left arm was noted. He was first presented to our Center for Chronically Sick Children at 2.6 years of age. He had spastic hemiparesis affecting particularly the left arm, hemineglect, and intermittent spontaneous nystagmus. A cranial MRI revealed a cystic parenchyma defect of the right parietotemporal side due to an ischemic stroke of his middle cerebral artery (Figure 1A). At this point, he had no reported seizures.

At the age of 9 years, the patient presented again because of global developmental delay, hemiparesis, and drug-refractory epilepsy with persistent seizures. ASM treatment with VPA (18.75 mg/kg/day) and lamotrigine (LTG) (0.95 mg/kg/day) failed to control the seizures. First focal-onset seizures had occurred at 4 years of age. Over time, these gradually shifted to focal-onset seizures with increasingly impaired awareness. At presentation, he had focal to tonic-clonic seizures every 8 weeks. He showed appropriate engagement during social contact with a slight decrease in proximity-distance regulation. In neuropsychological examination, difficulties in attentional control, impulse control, and inhibition, as well as slightly increased motor agitation could be observed. Furthermore, he showed reduced stress resilience and frustration tolerance in the presence of excessive emotions. Because of highly increased emotional stress of the parents, it was not possible to examine neuropsychological data *via* questionnaires.

We classified the patient as drug-resistant and included him in our epilepsy surgery program. Further investigations with congruent findings in MRI and EEG led to the indication for hemispherotomy. A vertical parasagittal hemispherotomy on the right side was performed without complications. Since then, the patient has remained seizure-free (Figure 1B).

**Figure 2:**
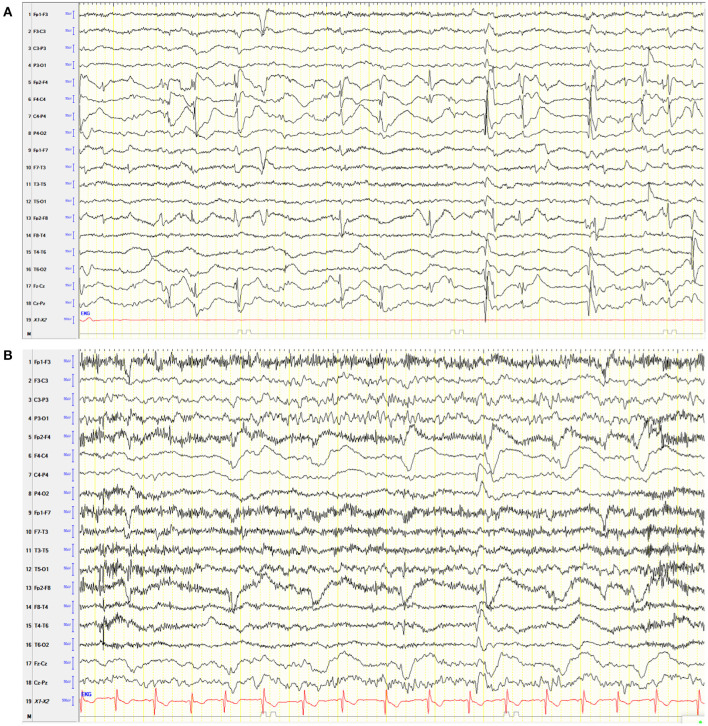
Electroencephalogram (EEG) of the patient. **(A)** Awake EEG after hemispherotomy under VPA. Hypersynchronous activity on the right frontocentral side over the disconnected hemisphere without evidence of clinical seizure signs and without evidence of transition to the left. **(B)** Six months after surgery without VPA, the EEG showed continuous right hemispheric dysfunction, hypersynchronic right frontocentral activity, and activation during sleep without clinical signs.

Two weeks after surgery, he showed an acute increase in psychological problems. He showed an acute aggravation of neuropsychological issues: hyperactivity, inhibition, and attentional control. The patient was restless and hardly controllable during medical examinations. Furthermore, the parents and teachers reported an increased tendency to aggressive behavior, which led to an exclusion from school. Overall, the symptoms had a high impact on the well-being of the family and the patient. An EEG showed no evidence of leftward transition with no evidence of clinical seizure signs (Figure 2A).

We considered these behavioral problems to be related to the postoperatively changed neuronal networks of the brain. This working hypothesis was based on clinical experience by one of the senior authors (CE) on VPA-induced behavioral disturbances after epilepsy surgery that has not been reported previously. Already after VPA reduction from 18.75 to 14 mg/kg/day, the behavioral problems decreased. Once VPA was tapered-off, the behavioral problems were back at the preoperative level. Serum levels of the ASM of the patient were not measured. An EEG performed 6 months post-operatively showed no evidence of seizures (Figure 2B). A standardized neuropsychological follow-up assessment 6, 12, and 24 months postoperatively revealed a significant improvement in attention span, inhibitory skills, and frustration tolerance. Aggressive behavior was not detected during the medical examinations. The parents reported them to be absent during everyday life.

## Discussion

Here, we report an acute aggravation of behavioral problems in a patient following hemispherotomy, which subsided after discontinuation of VPA. VPA is an inhibitor of γ amino butyric acid degradation and of voltage-gated ion channels. In addition to its use as an ASM, it is applied as a mood stabilizer to prevent aggressive impulsive behavior (3). However, behavioral abnormalities in association with VPA are known. They usually occur after drug initiation and, as in our case, disappear with tapering-off of the drug (4). In our case, however, the patient had been taking VPA for several years without severe behavioral problems. They aggravated acutely following hemispherotomy. There are no explanations in terms of an altered social environment or acute interpersonal experiences. Therefore, we propose VPA-induced behavioral problems due to postoperative altered neuronal network function and structures. We did not reintroduce the drug to test our hypothesis for ethical reasons. We did not measure VPA and LTG serum levels.

Behavioral problems after epilepsy surgery have been described. Although behavioral abnormalities are known to occur after surgery, in our patient, they disappeared when the drug dose was tapered and have not recurred since in a follow-up period of 30 months (5). We cannot rule out the spontaneous correction of post-hemispherotomy network dysregulation and, thus, a timely coincidence with the tapering of valproic acid. In adults and children, especially those with long-standing drug resistant epilepsy, there is a phenomenon referred to as forced normalization. Forced normalization is characterized by the occurrence of psychiatric problems after achieving seizure freedom (6). The underlying mechanisms are not well-understood. In forced normalization, however, symptoms typically persist for an average of 111 days and require antipsychotic therapy in about 73% of all patients. Control of symptoms is achieved in only 28.5% of patients with forced normalization after epilepsy surgery (6). This phenomenon is unlikely in our patient, since the symptoms reduced already after the reduction of the VPA dose by 4.75 mg/kg/day after 4 days, and no further therapy was needed.

It is known that the combination of LTG and VPA, although effective, also increases the risk of adverse effects, e.g., through an increase in LTG serum levels (7, 8). Both VPA and LTG have been associated with behavioral problems, and, therefore, both could be putatively responsible for the described symptoms (9). Tapering off of VPA led to the resolution of symptoms quickly, arguing more for a primary VPA effect given the shorter half-life of the ASM than for a primary LTG effect. In our patient, no severe adverse effects occurred preoperatively. Changes in ASM serum levels after epilepsy surgery have been reported temporary and directly after surgery (10, 11). Theoretically, a postoperative increase in VPA in our patient may have occurred and resulted in behavioral side effects. However, in our case, the symptoms were not present directly postoperatively. The patient rather presented more than 1 month after surgery with the described symptoms, rendering a surgery-related ASM serum level change unlikely. Moreover, the symptoms rapidly resolved after the reduction of VPA dose.

To further corroborate the proposed correlation between VPA-triggered behavioral abnormalities parallel to altered neuronal networks, we assessed the probability of an adverse drug reaction with the score presented in Koh et al. (12). The reaction is considered probable.

The aim of that report is to raise awareness to possible altered effects of VPA parallel to reorganizational processes after hemispherotomy. We could not find any description of acute behavioral issues after hemispherotomy in association with VPA in the PubMed research literature. Anticipating further reports, underlying pharmacological mechanisms should be studied. Furthermore, we highlight the importance of pre- and post-operative ASM levels to better assess such interactions.

## Data Availability Statement

The original contributions presented in the study are included in the article/supplementary material, further inquiries can be directed to the corresponding author.

## Ethics Statement

The studies involving human participants were reviewed and approved by Ethikkommission der Charité – Universitätsmedizin Berlin. Written informed consent for participation was not required for this study in accordance with the national legislation and the institutional requirements.

## Author Contributions

KM, CE, DA, and CP contributed to conception and design of the study. ST and KM organized the database and wrote the first draft of the manuscript. All authors contributed to manuscript revision, read, and approved the submitted version.

## Conflict of Interest

The authors declare that the research was conducted in the absence of any commercial or financial relationships that could be construed as a potential conflict of interest.

## Publisher's Note

All claims expressed in this article are solely those of the authors and do not necessarily represent those of their affiliated organizations, or those of the publisher, the editors and the reviewers. Any product that may be evaluated in this article, or claim that may be made by its manufacturer, is not guaranteed or endorsed by the publisher.
